# Supplementary material for: Mass-invariant universal optical conductivity from quantum geometry
Source: Sci Adv. 2026 Jun 5;12(23):eady2033. doi: 10.1126/sciadv.ady2033 (PMC13267284; doi:10.1126/sciadv.ady2033)
Supplement: Supplementary file 1 — Supplementary Text S1 to S12 Figs. S1 and S2 Table S1 References [file sciadv.ady2033_sm.pdf]

Science Advances
AAAS

Supplementary Materials for

Mass-invariant universal optical conductivity from quantum geometry

Chang-geun Oh *et al.*

Corresponding author: Chang-geun Oh, cg.oh.0404@gmail.com; Sun-Woo Kim, sunwookim@hanyang.ac.kr; Kun Woo Kim, kunx@cau.ac.kr; Bartomeu Monserrat, bm418@cam.ac.uk; Jun-Won Rhim, jwrhim@ajou.ac.kr

Sci. Adv. **12**, eady2033 (2026)
DOI: 10.1126/sciadv.ady2033

This PDF file includes:

Supplementary Text S1 to S12
Figs. S1 and S2
Table S1
References

I. OPTICAL CONDUCTIVITY FROM KUBO FORMULA

For the convenience of readers, we review the optical conductivity obtained from Kubo formula. According to the Kubo formula, the change of physical observable B from the one in thermal equilibrium is given by

$$\Delta B(t) = \frac{i}{\hbar}\int_0^\infty dt' F(t-t')\left\langle\left[\hat{B}(t'),\hat{A}\right]\right\rangle, \tag{S1}$$

when the external Hamiltonian is $\hat{H}'(t) = -F(t)\hat{A}$. Here, we consider the case with $\hat{H}'(t) = -\hat{\boldsymbol{x}}\cdot\boldsymbol{E}e^{-i\omega t}$ and $\hat{B} = \hat{J}_{i,s_\nu} = \frac{1}{2}\left\{\frac{\partial \hat{H}}{\partial k_i}, \sigma_\nu\right\} = \frac{1}{2}\left(\hat{J}_{i,s_0}\sigma_\nu + \sigma_\nu\hat{J}_{i,s_0}\right)$, where $i = x, y$ and $\nu = 0, x, y, z$. The charge current density is obtained for $\nu = 0$, while the spin current densities for the x, y, and z spin components are determined for $\nu = x$, y, and z, respectively. For simplicity, we use $e = \hbar = 1$ unit. Then, the current density J_{i,s_ν} is given by

$$J_{i,s_\nu}(\omega) = i\int_0^\infty dt' \sum_j e^{i\omega t'}\left\langle\left[\hat{J}_{i,s_\nu}(t'),\hat{x}_j\right]\right\rangle E_j e^{-i\omega t} \tag{S2}$$

and the conductivity is

$$\sigma_{ij}^{\nu}(\omega) = i\int_0^\infty dt e^{i\omega t-\delta t}\left\langle\left[\hat{J}_{i,s_\nu}(t),\hat{x}_j\right]\right\rangle \tag{S3}$$

$$= i\int_0^\infty dt e^{i\omega t-\delta t}\left\langle \hat{J}_{i,s_\nu}(t)\hat{x}_j - \hat{x}_j\hat{J}_{i,s_\nu}(t)\right\rangle \tag{S4}$$

$$= i\int_0^\infty dt \sum_{m,n} e^{i\omega t-\delta t}\left(f_n\langle n|\hat{J}_{i,s_\nu}(t)\hat{x}_j|n\rangle - f_m\langle m|\hat{x}_j\hat{J}_{i,s_\nu}(t)|m\rangle\right) \tag{S5}$$

$$= i\int_0^\infty dt \sum_{m,n} e^{i\omega t-\delta t}\left(f_n\langle n|\hat{J}_{i,s_\nu}(t)|m\rangle\langle m|\hat{x}_j|n\rangle - f_m\langle m|\hat{x}_j|n\rangle\langle n|\hat{J}_{i,s_\nu}(t)|m\rangle\right) \tag{S6}$$

$$= i\int_0^\infty dt \sum_{m,n} e^{i\omega t-\delta t}\left(f_n\langle n|e^{i\hat{H}t}\hat{J}_{i,s_\nu}e^{-i\hat{H}t}|m\rangle\langle m|\hat{x}_j|n\rangle - f_m\langle m|\hat{x}_j|n\rangle\langle n|e^{i\hat{H}t}\hat{J}_{i,s_\nu}e^{-i\hat{H}t}|m\rangle\right) \tag{S7}$$

$$= i\int_0^\infty dt \sum_{m,n} e^{i(\omega+\epsilon_n-\epsilon_m)t-\eta t}\left(f_n\langle n|\hat{J}_{i,s_\nu}|m\rangle\langle m|\hat{x}_j|n\rangle - f_m\langle m|\hat{x}_j|n\rangle\langle n|\hat{J}_{i,s_\nu}|m\rangle\right) \tag{S8}$$

$$= \sum_{m,n}\frac{-1}{\omega+\epsilon_n-\epsilon_m+i\eta}\left(f_n\langle n|\hat{J}_{i,s_\nu}|m\rangle\langle m|\hat{x}_j|n\rangle - f_m\langle m|\hat{x}_j|n\rangle\langle n|\hat{J}_{i,s_\nu}|m\rangle\right) \tag{S9}$$

Using

$$\langle n(\boldsymbol{k})|\hat{x}_j|m(\boldsymbol{k})\rangle = \frac{1}{i(\epsilon_n(\boldsymbol{k})-\epsilon_m(\boldsymbol{k}))}\langle u_n(\boldsymbol{k})|\hat{J}_j|u_m(\boldsymbol{k})\rangle, \tag{S10}$$

we get

$$\sigma_{ij}^{\nu}(\omega) = \frac{1}{i}\int\frac{d^2\boldsymbol{k}}{(2\pi)^2}\sum_{m,n}\frac{f(\epsilon_n(\boldsymbol{k}))-f(\epsilon_m(\boldsymbol{k}))}{\epsilon_n(\boldsymbol{k})-\epsilon_m(\boldsymbol{k})}\frac{\langle n\boldsymbol{k}|\hat{J}_{i,s_\nu}|m\boldsymbol{k}\rangle\langle m\boldsymbol{k}|\hat{J}_{j,s_0}|n\boldsymbol{k}\rangle}{\omega+\epsilon_n(\boldsymbol{k})-\epsilon_m(\boldsymbol{k})+i\delta}, \tag{S11}$$

where $f(\epsilon)$ is the Fermi-Dirac distribution. For $\nu = 0$, using

$$\langle u_n(\boldsymbol{k})|\hat{J}_j|u_m(\boldsymbol{k})\rangle = i(\epsilon_m(\boldsymbol{k}) - \epsilon_n(\boldsymbol{k}))A^j_{nm} + \epsilon_{k_x}\epsilon_{n,\boldsymbol{k}}\delta_{nm}, \tag{S12}$$

we obtain

$$\sigma_{ij}(\omega) = \int \frac{d^2k}{(2\pi)^2} \sum_{n,m} F_{nm}(\boldsymbol{k}) \frac{i\epsilon_{mn}(\boldsymbol{k})A^i_{nm}(\boldsymbol{k})A^j_{mn}(\boldsymbol{k})}{\epsilon_{nm}(\boldsymbol{k}) + \omega + i\eta} + \sigma^{\text{intra}}_{ij}(\omega), \tag{S13}$$

where

$$\sigma^{\text{intra}}_{ij}(\omega) = i \int \frac{d^2k}{(2\pi)^2} \sum_{n} \left(-\frac{\partial f(\epsilon_{n,\boldsymbol{k}})}{\partial \epsilon_{n,\boldsymbol{k}}} \right) \frac{(v_{n,\boldsymbol{k},i})(v_{n,\boldsymbol{k},j})}{\omega + i\eta}. \tag{S14}$$

Here, $v_{n,\boldsymbol{k},j} = \partial_{k_j}\epsilon_{n,\boldsymbol{k}}$ is the group velocity along the j-direction.

II. QUANTUM GEOMETRIC TENSOR AND LINEAR OPTICAL RESPONSES

The quantum geometric tensor can be expressed in terms of multi-band Berry connections:

$$\mathcal{G}^n_{ij} = = \left\langle \frac{\partial \psi_n}{\partial \Lambda_i} \middle| \sum_{m\neq n} |\psi_m\rangle\langle\psi_m| \middle| \frac{\partial \psi_n}{\partial \Lambda_j} \right\rangle = \sum_{m\neq n} \langle \frac{\partial \psi_n}{\partial \Lambda_i} |\psi_m\rangle\langle\psi_m| \frac{\partial \psi_n}{\partial \Lambda_i} \rangle, \tag{S15}$$

$$= \sum_{m\neq n} A^i_{nm}(A^j_{nm})^* = \sum_{m\neq n} \frac{A^i_{nm}(A^j_{nm})^* + A^j_{nm}(A^i_{nm})^*}{2} + \frac{A^i_{nm}(A^j_{nm})^* - A^j_{nm}(A^i_{nm})^*}{2}, \tag{S16}$$

$$\equiv \sum_{m\neq n} g^{nm}_{ij}(\mathbf{\Lambda}) - \frac{i}{2}\Omega^{nm}_{ij}(\mathbf{\Lambda}). \tag{S17}$$

where $\hat{I} = \sum_m |\psi_m\rangle\langle\psi_m|$ is used in the first line, and the inter-band quantum metric tensor and the Berry curvature are introduced as

$$g^{nm}_{ij}(\mathbf{\Lambda}) = \frac{A^i_{nm}(A^j_{nm})^* + A^j_{nm}(A^i_{nm})^*}{2}, \tag{S18}$$

$$\Omega^{nm}_{ij}(\mathbf{\Lambda}) = i\left[A^i_{nm}(A^j_{nm})^* - A^j_{nm}(A^i_{nm})^*\right]. \tag{S19}$$

Note that for $i = j$, the antisymmetric component $\Omega^{nm}_{ii} = 0$. The optical conductivity expressed in terms of multi-band Berry connections is then naturally expressed in terms of multi-band quantum geometric tensors:

$$\sigma_{ij}(\omega) = \int \frac{d^2k}{(2\pi)^2} \sum_{n,m} F_{nm}(\boldsymbol{k}) \frac{i\epsilon_{mn}(\boldsymbol{k}) A^i_{nm}(\boldsymbol{k})(A^j_{nm}(\boldsymbol{k}))^*}{\epsilon_{nm}(\boldsymbol{k}) + \omega + i\eta}, \tag{S20}$$

$$= \int \frac{d^2k}{(2\pi)^2} \sum_{n,m} F_{nm}(\boldsymbol{k}) \frac{i\epsilon_{mn}(\boldsymbol{k})}{\epsilon_{nm}(\boldsymbol{k}) + \omega + i\eta} \left[g^{nm}_{ij}(\boldsymbol{k}) - \frac{i}{2}\Omega^{nm}_{ij}(\boldsymbol{k})\right], \tag{S21}$$

which implies an optical longitudinal conductivity $\sigma_{ii}(\omega)$ contains in general the information of multi-band quantum metric tensor g^{nm}_{ii}, and an optical Hall conductivity $\sigma_{xy}(\omega)$ contains additional contribution from the Berry curvature.

In two-band system with $\eta \to 0$, a straightforward calculation leads to

$$\mathrm{Re}[\sigma_{ij}(\omega)] = \pi \int \frac{d^2\boldsymbol{k}}{(2\pi)^2} F_{lu}(\boldsymbol{k})\epsilon_{ul}(\boldsymbol{k}) g^u_{ij}(\boldsymbol{k}) \delta(\omega - \epsilon_{ul}), \tag{S22}$$

where u and l denote upper and lower bands, respectively. This equation shows that the optical conductivity is determined by the band dispersions $\epsilon_n(\boldsymbol{k})$ and quantum metric tensor $g^u_{ij}(\boldsymbol{k})$. For the 2-dimensional quadratic band touching model, $\sigma_{ii}(\omega)$ is independent of driving frequency ω as well as mass $m_{u,l}$.

III. GENERAL FORM OF THE ISOTROPIC BAND TOUCHING MODEL

In this section, we summarize the derivation, following Ref. [34], of the general two-dimensional quadratic band-touching (QBT) Hamiltonian that yields isotropic dispersions

$$\epsilon_{u/l} = \frac{k^2}{2m_{u/l}}.$$

Such a model can be parametrized entirely by the band masses m_u, m_l and the maximal quantum distance $d_{\max}$.

The generic QBT Hamiltonian in the Pauli matrix basis reads [54]

$$\mathcal{H}(\boldsymbol{k}) = \sum_{\alpha=0,x,y,z} h_\alpha(\boldsymbol{k}), \sigma_\alpha, \tag{S23}$$

where the real functions $h_\alpha(\boldsymbol{k})$ are quadratic in momentum:

$$\begin{aligned} h_0(\boldsymbol{k}) &= b_1 k_x^2 + b_2 k_x k_y + b_3 k_y^2, \\ h_x(\boldsymbol{k}) &= t_6 k_y^2, \\ h_y(\boldsymbol{k}) &= t_4 k_x k_y + t_5 k_y^2, \\ h_z(\boldsymbol{k}) &= t_1 k_x^2 + t_2 k_x k_y + t_3 k_y^2. \end{aligned} \tag{S24}$$

Without loss of generality we set $t_4 > 0$, since its sign does not affect the resulting optical conductivity. Imposing the target dispersions

$$h_0^2 = |\boldsymbol{k}|^4 \frac{1}{16}\left(\frac{1}{m_+} + \frac{1}{m_-}\right)^2, \quad h_x^2 + h_y^2 + h_z^2 = |\boldsymbol{k}|^4 \frac{1}{16}\left(\frac{1}{m_+} - \frac{1}{m_-}\right)^2 \tag{S25}$$

yields algebraic constraints that fix all nine coefficients in terms of m_u, m_l and $d_{\max}$.

$$\begin{aligned} t_1 &= \frac{1}{4}\left(\frac{1}{m_u} - \frac{1}{m_l}\right), \;\; t_2 = 0, \;\; t_3 = \frac{1 - 2d_{\max}^2}{4}\left(\frac{1}{m_u} - \frac{1}{m_l}\right), \\ t_4 &= \frac{d_{\max}}{2}\left(\frac{1}{m_u} - \frac{1}{m_l}\right), \;\; t_5 = 0, \;\; t_6 = \frac{d_{\max}\sqrt{1 - d_{\max}^2} k_y^2}{2}\left(\frac{1}{m_u} - \frac{1}{m_l}\right), \\ b_1 &= \frac{1}{4}\left(\frac{1}{m_u} + \frac{1}{m_l}\right), \;\; b_2 = 0, \;\; b_3 = \frac{1}{4}\left(\frac{1}{m_u} + \frac{1}{m_l}\right). \end{aligned} \tag{S26}$$

These expressions demonstrate that the isotropic QBT model is fully determined by three continuous parameters $(m_+, m_-, d_{\max})$.

IV. DERIVATION OF THE UNIVERSAL OPTICAL CONDUCTIVITY

In this section, we derive the universal optical conductivity. The Pauli blocking condition from F_{lu} in Eq. (S22) will be applied at the end.

For the given model, the band energy difference is $\epsilon_{ul}(\boldsymbol{k}) = \epsilon_u - \epsilon_l = (1/2m_u - 1/2m_l)k^2 = k^2/(2M)$. The quantum metric tensor components for the upper band are

$$g^u_{xx}(\boldsymbol{k}) = d^2_{\max}\frac{k_y^2}{k^4}, \quad g^u_{yy}(\boldsymbol{k}) = d^2_{\max}\frac{k_x^2}{k^4}, \quad g^u_{xy}(\boldsymbol{k}) = -d^2_{\max}\frac{k_x k_y}{k^4}. \tag{S27}$$

Substituting these into Eq. (S22) and converting to polar coordinates ($k_x = k\cos\theta, k_y = k\sin\theta, d^2\boldsymbol{k} = k\,dk\,d\theta$):

$$\text{Re}[\sigma_{ij}(\omega)] = \frac{\pi}{4\pi^2}\int_0^{2\pi} d\theta \int_0^{\infty} k\,dk \left(\frac{k^2}{2M}\right) g^u_{ij}(k,\theta)\delta\left(\omega - \frac{k^2}{2M}\right). \tag{S28}$$

Let's evaluate the diagonal component $\text{Re}[\sigma_{xx}(\omega)]$, where $g^u_{xx} = (d^2_{\max}/k^2)\sin^2\theta$:

$$\text{Re}[\sigma_{xx}(\omega)] = \frac{d^2_{\max}}{4\pi M}\int_0^{2\pi}\sin^2\theta\,d\theta\int_0^{\infty} k\,\delta\left(\omega - \frac{k^2}{2M}\right)dk. \tag{S29}$$

The Dirac delta function fixes the resonant momentum at $k_0 = \sqrt{2M\omega}$. The integral over k evaluates to:

$$\int_0^{\infty} k\,\delta\left(\hbar\omega - \frac{k^2}{2M}\right)dk = \left|\frac{dk}{d(k^2/2M)}\right|_{k=k_0} k_0 = M. \tag{S30}$$

The angular integral is $\int_0^{2\pi}\sin^2\theta\,d\theta = \pi$. Combining these results:

$$\text{Re}[\sigma_{xx}(\omega)] = \frac{1}{8}d^2_{\max}. \tag{S31}$$

A similar calculation for the yy-component using $\int_0^{2\pi}\cos^2\theta\,d\theta = \pi$ yields an identical result. The off-diagonal xy-component vanishes because $\int_0^{2\pi}\cos\theta\sin\theta\,d\theta = 0$. Thus, the tensor is diagonal.

Finally, we apply the Pauli blocking principle. For a transition to occur, the final state energy must be above the chemical potential μ: $\epsilon_u(k_0) > \mu$.

$$\epsilon_u(k_0) = \frac{k_0^2}{2m_u} = \frac{2M\omega}{2m_u} = \frac{M}{m_u}\omega > \mu. \tag{S32}$$

This imposes a condition on the frequency: $\omega > \frac{m_u}{M}\mu \equiv \mu^*$. This is represented by the Heaviside step function $\Theta(\hbar\omega - \mu^*)$.

The complete expression for the optical conductivity is:

$$\text{Re}[\sigma_{ij}(\omega)] = \frac{d^2_{\max}}{8}\delta_{ij}\Theta(\omega - \mu^*). \tag{S33}$$

V. KAGOME LATTICE MODEL

In the main text, we calculated the optical conductivity of the Kagome lattice model. We only consider the nearest neighbor hopping processes. The Hamiltonian in momentum space is given by

$$H^{\text{Kagome}} = \begin{pmatrix} 2 & e^{-i\boldsymbol{a}_1\cdot\boldsymbol{k}/2}(e^{-i\boldsymbol{a}_1\cdot\boldsymbol{k}}+1) & e^{-i\boldsymbol{a}_2\cdot\boldsymbol{k}/2}(e^{-i\boldsymbol{a}_2\cdot\boldsymbol{k}}+1) \\ e^{+i\boldsymbol{a}_1\cdot\boldsymbol{k}/2}(e^{-i\boldsymbol{a}_1\cdot\boldsymbol{k}}+1) & 2 & e^{+i(\boldsymbol{a}_1-\boldsymbol{a}_2)\cdot\boldsymbol{k}/2}(e^{-i\boldsymbol{a}_3\cdot\boldsymbol{k}}+1) \\ e^{+i\boldsymbol{a}_2\cdot\boldsymbol{k}/2}(e^{-i\boldsymbol{a}_2\cdot\boldsymbol{k}}+1) & e^{-i(\boldsymbol{a}_1-\boldsymbol{a}_2)\cdot\boldsymbol{k}/2}(e^{-i\boldsymbol{a}_3\cdot\boldsymbol{k}}+1) & 2 \end{pmatrix}, \tag{S34}$$

where $\boldsymbol{a_1} = (1,0)$, $\boldsymbol{a_2} = (-1/2, \sqrt{3}/2)$, and $\boldsymbol{a_3} = (-1/2, -\sqrt{3}/2)$. The lowest two band dispersions are illustrated in Fig. 2**c** of the main text. This induces a flat band at $E = 0$, a parabolic band having a band touching at $\boldsymbol{k} = 0$ with $m_u^{-1} = 1/2$ and $d_{\max} = 1$.

VI. HIGHER ORDER EFFECTS ON OPTICAL CONDUCTIVITY

Small deviations from $d_{\mathrm{max}}^2/8$ in optical conductivity, which disrupt the flatness of the optical response, are observed in both the tight-binding model and DFT calculations (see Figs. 2**b,d**, 3**f,i,l**, and 4**d** in the main text). In this section, we numerically demonstrate that these deviations can be attributed to higher-order effects, providing an illustrative example.

We consider the following model:

$$\mathcal{H} = \mathcal{H}_0(\boldsymbol{k}) + \mathcal{H}_{\mathrm{pert}}, \tag{S35}$$

where $\mathcal{H}_0$ is the Hamiltonian in Eq. (12) of the main text, and $\mathcal{H}_{\mathrm{pert}} = a(k_x^4 + k_y^4)\sigma_3$, which introduces higher order effects to the energy and optical conductivity. Supplementary Figs. S1**a** and **b** show the band dispersion of the upper band at $k_y = 0$ and the optical conductivity for $d_{\mathrm{max}} = 1$. The black, red, and blue plots correspond to $a = 0$, -0.1, and 0.1, respectively. When $a \neq 0$, deviations from the flat optical conductivity at 1/8. are observed. This indicates that higher-order effects are responsible for these deviations.

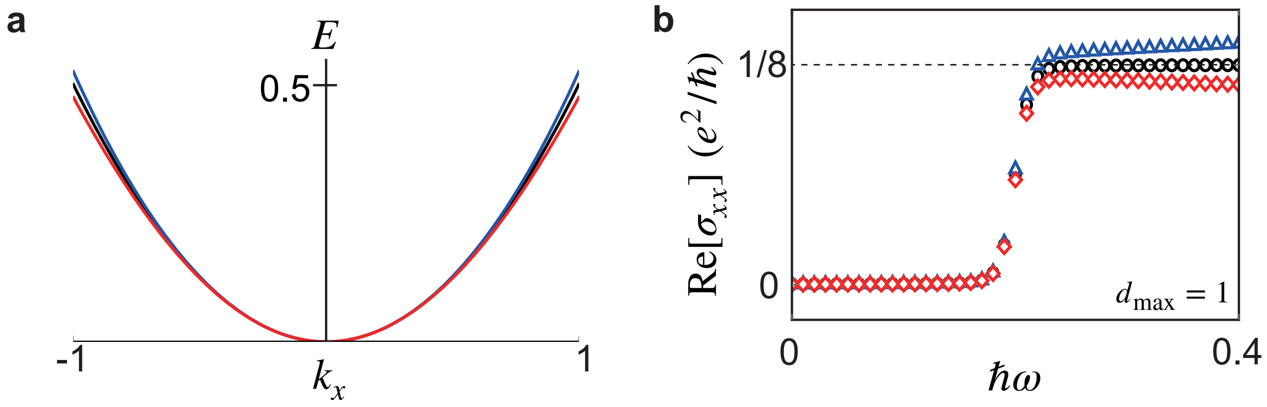

Fig. S1. **Higher order corrections of energy and optical conductivity.** **a** Band dispersions of the upper band at $k_y = 0$. **b** Frequency ω dependence of the real part of the optical conductivity $Re[\sigma_{xx}]$. In **a** and **b**, the black, red, and blue plots correspond to $a = 0$, -0.1, and 0.1, respectively. Here, $m_u = -m_l = 1$, $\mu^* = 0.2$ and $T = 0.003$ are used.

VII. GAP EFFECTS ON OPTICAL CONDUCTIVITY

In this section, we investigate the effects of a band gap on the optical conductivity.

We consider the following model:

$$\mathcal{H} = \mathcal{H}_0(\boldsymbol{k}) + \mathcal{H}_{\text{gap}}, \tag{S36}$$

where $\mathcal{H}_0$ is the Hamiltonian given in Eq. (12) of the main text, and $\mathcal{H}_{\text{gap}} = \sum_{i=x,y,z} \Delta_i \sigma_i$, which introduces a small gap ($\Delta_i \ll 1$) at the band touching. The energy dispersion is given by:

$$\begin{aligned} E_{\pm}^{\Delta} =& \frac{k^2}{4}(m_r^{-1} + 2m_l^{-1}) \\ &\pm \frac{1}{4}\sqrt{\frac{k^4}{m_r} + \frac{8}{m_r}\left\{2d_{\max}k_y(\sqrt{1-d_{\max}^2}k_y\Delta_x + k_x\Delta_y) - 2d_{\max}^2 k_y^2\Delta_z + k^2\Delta_z\right\} + 16\Delta^2}, \end{aligned} \tag{S37}$$

where $\Delta^2 = \Delta_x^2 + \Delta_y^2 + \Delta_z^2$. A straightforward calculation yields the following expression for the optical conductivity

$$\mathrm{Re}\left[\sigma_{xx}(\omega > 2|\Delta|)\right] \approx \frac{d_{\max}^2}{8} + \Delta_x \frac{d_{\max}\sqrt{1-d_{\max}^2}(1-3d_{\max}^2)}{4\omega} - \Delta_z \frac{3d_{\max}^2(1-d_{\max}^2)}{2\omega} + O\left(\frac{\Delta_i^2}{\omega^2}\right). \tag{S38}$$

This result demonstrates that the universal optical conductivity remains robust against small band gap openings. Furthermore, for $d_{\max} = 1$, the deviation from $1/8$ begins at $O(\Delta_i^2/\omega^2)$, indicating an even stronger robustness, as exemplified in the honeycomb Bi monolayer discussed in the main text.

VIII. QUANTUM METRIC FOR DIRAC MASSIVE AND QUADRATIC MASSIVE MODELS

The quantum metric for a massive Dirac fermion varies with changes in its mass. In contrast, for the quadratic band touching model, the quantum metric is independent of mass. In this section, we provide a detailed demonstration of this distinction.

In a two-band system, the Hamiltonian is expressed as

$$H(\boldsymbol{k}) = h_0(\boldsymbol{k}) + \boldsymbol{h}(\boldsymbol{k}) \cdot \boldsymbol{\sigma}, \tag{S39}$$

where $\boldsymbol{\sigma} = (\sigma_x, \sigma_y, \sigma_z)$ denotes the Pauli matrices. The pseudospin of the upper band is characterized as $\boldsymbol{s} = \boldsymbol{h}/|\boldsymbol{h}|$. Using this pseudospin, the geometric quantities can be written as

$$g^n_{ij}(\boldsymbol{k}) = \frac{1}{4}\partial_{k_i}\boldsymbol{s}(\boldsymbol{k}) \cdot \partial_{k_j}\boldsymbol{s}(\boldsymbol{k}), \tag{S40}$$

where n is the band index; $n = u$ denotes the upper band and $n = l$ the lower band.

The massive Dirac fermion model is described by

$$H_{\mathrm{D}} = v(k_x\sigma_x + k_y\sigma_y) + m\sigma_z, \tag{S41}$$

where m is the mass and v is the velocity of the Dirac fermion. The energy dispersion is given by $E_\pm = \pm\sqrt{v^2k^2 + m^2}$. A straightforward calculation yields the quantum metric components:

$$g^n_{xx}(\boldsymbol{k}) = \frac{v^2(m^2 + v^2k_y^2)}{4E_\pm^4},\ \ g^n_{yy}(\boldsymbol{k}) = \frac{v^2(m^2 + v^2k_x^2)}{4E_\pm^4},\ \ g^n_{xy}(\boldsymbol{k}) = g^n_{yx}(\boldsymbol{k}) = -\frac{v^4(k_xk_y)}{4E_\pm^4}. \tag{S42}$$

These results confirm that the quantum geometric is directly influenced by the mass.

In contrast, the quadratic band touching model, as defined in Eq. (12) of the main text, is expressed as

$$\mathcal{H}_0(\boldsymbol{k}) = \sum_\alpha h_\alpha(\boldsymbol{k})\sigma_\alpha, \tag{S43}$$

where σ_α represents an identity ($\alpha = 0$) and Pauli matrices ($\alpha = x, y, z$). The components $h_\alpha(\boldsymbol{k})$ are real quadratic functions: $h_0(\boldsymbol{k}) = (1/m_r + 2/m_l)(k_x^2 + k_y^2)/4$, $h_x(\boldsymbol{k}) = d_{\max}\sqrt{1 - d_{\max}^2}k_y^2/(2m_r)$, $h_y(\boldsymbol{k}) = d_{\max}k_xk_y/(2m_r)$, and $h_z(\boldsymbol{k}) = (k_x^2 + (1 - 2d_{\max}^2)k_y^2)/(4m_r)$, where $1/m_r = 1/m_u - 1/m_l$. A straightforward calculation leads to the quantum metric components:

$$g^n_{xx}(\boldsymbol{k}) = d_{\max}^2\frac{k_y^2}{k^4},\ \ g^n_{yy}(\boldsymbol{k}) = d_{\max}^2\frac{k_x^2}{k^4},$$
$$g^n_{xy}(\boldsymbol{k}) = g^n_{yx}(\boldsymbol{k}) = -d_{\max}^2\frac{k_xk_y}{k^4}. \tag{S44}$$

These results reveal that the quantum metric is independent of the mass in this model.

Table S1. Comparison of the quantum metric between the massive Dirac fermion and quadratic band touching models.

**Aspect**	**Massive Dirac Fermion Model**	**Quadratic Band Touching Model**
Energy	$E_{\pm} = \pm\sqrt{v^2k^2 + m^2}$	$\epsilon_{u/l} = k^2/(2m_{u/l})$
Quantum Metric	$g^n_{xx} = \frac{v^2(m^2+v^2k_y^2)}{4E_{\pm}^4}$	$g^n_{xx} = d^2_{\max}\frac{k_y^2}{k^4}$
	$g^n_{yy} = \frac{v^2(m^2+v^2k_x^2)}{4E_{\pm}^4}$	$g^n_{yy} = d^2_{\max}\frac{k_x^2}{k^4}$
	$g^n_{xy} = g^n_{yx} = -\frac{v^4k_xk_y}{4E_{\pm}^4}$	$g^n_{xy} = g^n_{yx} = -d^2_{\max}\frac{k_xk_y}{k^4}$
Note	Quantum metric depends on mass	Quantum metric is independent of mass

IX. SYMMETRY CONSTRAINTS ON THE HILBERT-SCHMIDT QUANTUM DISTANCE

In this section, we examine the influence of C_n-rotational symmetry and time-reversal symmetry on the wave function geometry within the framework of the quadratic band-touching model. In particular, we build upon and apply the findings from previous works [53,55,74] to our model.

For the spinless system, the C_n-rotation operator satisfies $(C_n)^n = 1$. For a two-band system, the C_n operator can be represented as:

$$C_n = \begin{pmatrix} e^{i\frac{2\pi a}{n}} & 0 \\ 0 & e^{i\frac{2\pi b}{n}} \end{pmatrix}, \quad (a, b = 0, \dots, n-1). \tag{S45}$$

Representations with (a, b) and (a', b') are equivalent up to a unitary transformation or a $U(1)$ phase, provided that $a - b = \pm(a' - b') \pmod n$. A generic Hamiltonian for a two-band system can be expressed as:

$$H(\boldsymbol{k}) = \sum_{l=0,3,\pm} h_l(\boldsymbol{k})\sigma_l, \tag{S46}$$

where $k_\pm = k_x \pm ik_y$ and $\sigma_\pm = \frac{1}{2}(\sigma_x \pm i\sigma_y)$. Here, $h_0(\boldsymbol{k})$ and $h_3(\boldsymbol{k})$ are real functions of $\boldsymbol{k}$, while $h_\pm(\boldsymbol{k})$ are complex functions satisfying $h_\pm(\boldsymbol{k}) = h^*_\mp(\boldsymbol{k})$. Each $h_a(\boldsymbol{k})$ can be expanded as a power series in $k_\pm$:

$$h_0(\boldsymbol{k}) = \sum_{l,j\geq 0} A_{lj} k_+^l k_-^j, \quad h_3(\boldsymbol{k}) = \sum_{l,j\geq 0} B_{lj} k_+^l k_-^j, \quad h_+(\boldsymbol{k}) = \sum_{l,j\geq 0} C_{ij} k_+^l k_-^j, \tag{S47}$$

where $A_{ij}, B_{ij} \in \mathbb{R}$ and $C_{ij} \in \mathbb{C}$. The symmetry condition $C_n H(\boldsymbol{k}) C_n^{-1} = H(C_n\boldsymbol{k})$ imposes the following constraints:

$$\begin{aligned} h_{0,3}(k_+, k_-) &= h_{0,3}(e^{i\frac{2\pi}{n}}k_+, e^{-i\frac{2\pi}{n}}k_-), \\ h_+(k_+, k_-) &= e^{-i\frac{2\pi(a-b)}{n}} h_+(e^{i\frac{2\pi}{n}}k_+, e^{-i\frac{2\pi}{n}}k_-). \end{aligned} \tag{S48}$$

As a result, $A_{lj} = B_{lj} = 0$ if $l - j \notin n\mathbb{Z}$, and $C_{lj} = 0$ if $l - j - (a - b) \notin n\mathbb{Z}$. To second order in $\boldsymbol{k}$, $h_0(\boldsymbol{k})$ and $h_3(\boldsymbol{k})$ can be simplified as:

$$h_0(\boldsymbol{k}) = A_{00} + A_{11}k^2, \quad h_3(\boldsymbol{k}) = B_{00} + B_{11}k^2, + B_{20}(k_x^2 - k_y^2)\delta_{n,2} \tag{S49}$$

where this form holds regardless of the specific values of a and b. In our model, $A_{00} = B_{00} = 0$. The terms allowed for h_+ depend on the symmetry C_n and the difference $a - b$. The allowed terms are summarized

in the following table:

n	$a-b$	$h_+(k_+,k_-)$
2	0	$C_{11}k_+k_- + C_{20}k_+^2 + C_{02}k_-^2$
2	1	0
3	0	$C_{11}k_+k_-$
3	1	$C_{20}k_-^2$
4	0	$C_{11}k_+k_-$
4	1	0
4	2	$C_{20}k_+^2 + C_{02}k_-^2$
6	0	$C_{11}k_+k_-$
6	1	0
6	2	$C_{20}k_+^2$
6	3	0

.

Next, we analyze time-reversal symmetry T, which satisfies $T^2 = 1$ in spinless systems and commutes with C_n symmetry. In the basis where the representation of C_n is diagonal, T can be represented as either K or $\sigma_x K$, where K denotes the complex conjugation operator.

- Case 1: $T = K$. In this scenario, time reversal symmetry enforces σ_y coefficients to remain zero. Thus, the allowed terms are summarized in the following:

n	$a-b$	$h_+(k_+,k_-)$
2	0	$C_{20}(k_x^2 - k_y^2)$
2	1	0
3	0	0
3	1	0
4	0	0
4	1	0
4	2	$C_{20}(k_x^2 - k_y^2)$
6	0	0
6	1	0
6	2	0
6	3	0

. Note that the C_{11} term can be eliminated through a unitary transformation $U = e^{-i\phi\sigma_y/2}$, where $\phi = \arctan(-C_{11}/B_{11})$, with a suitable redefinition of the coefficients. Therefore, we omitted the C_{11} term in the table. The intersection of all cases and the isotropic QBT model is only when $d_{\max} = 0$ with $C_{20} = B_{20} = 0$.

- Case 2: $T = \sigma_x K$. Time-reversal symmetry imposes $(a, b) = (a, -a)$ and $h_3(\boldsymbol{k}) = -h_3(-\boldsymbol{k})$, leading to $h_3(\boldsymbol{k}) = 0$ for quadratic Hamiltonians. The allowed terms and $d_{\max}$ are:

n	$a-b$	$h_+(k_+, k_-)$	$d_{\max}$
2	0	$C_{11}k^2 + C_{20}k_+^2 + C_{02}k_-^2$	$0-1$
2	1	$-$	$-$
3	0	$C_{11}k^2$	0
3	1	$C_{20}k_-^2$	1
4	0	$C_{11}k^2$	0
4	1	$-$	$-$
4	2	$C_{20}k_+^2 + C_{02}k_-^2$	1
6	0	$C_{11}k^2$	0
6	1	$-$	$-$
6	2	$C_{20}k_+^2$	1
6	3	$-$	$-$

For all cases, the isotropic condition only allows $d_{\max} = 0$ or 1.

Thus, when both T and C_n symmetries under isotropic condition are present, our model permits only two values: $d_{\max} = 0$ or $d_{\max} = 1$.

Note that these symmetry considerations may not be generally applicable to systems where spin-orbit coupling (SOC) is strong, as spin effects become physically significant. However, for nonmagnetic systems, such as in the Bi case in the main text, the spin degrees of freedom do not need to be explicitly considered, despite the strong SOC. In the Bi calculation presented in the main text, due to the presence of both inversion and time-reversal symmetries, the spin degrees of freedom can be effectively omitted from explicit consideration. Consequently, the essential physics can be described without directly involving spin, and $d_{\max} = 1$ arises from the combined C_3 and T symmetries, as manifested in the universal optical conductivity of $1/8(e^2/\hbar)$ for the Bi case, as displayed in the main text.

X. ANISOTROPIC CASE WITH SYMMETRY CONSTRAINTS

In the main text, we consider the quadratic band-touching (QBT) model under isotropic conditions. However, the isotropic condition is not always guaranteed. We investigate the optical conductivity for anisotropic QBT models under C_2 and T symmetries, or C_4 and T symmetries. Our focus remains on QBT systems.

We first focus on $T = \sigma_x K$ case. Note that this representation is equivalent to

$$C_n = \begin{pmatrix} \cos\frac{2\pi a}{n} & -\sin\frac{2\pi a}{n} \\ \sin\frac{2\pi a}{n} & \cos\frac{2\pi a}{n} \end{pmatrix}, \quad T = K, \tag{S50}$$

up to a unitary transformation. When C_n ($n = 3, 6$) and $T = \sigma_x K$ symmetries are present, only isotropic QBT models with $d_{\max} = 0$ or 1 are allowed. However, under C_n ($n = 2, 4$) and $T = \sigma_x K$ symmetries, the following Hamiltonian is allowed (see the previous section for details):

$$H_{\text{aniso1}} = a_0 k^2 \sigma_0 + a_x(k_x^2 - k_y^2)\sigma_x + 2a_{xy}k_x k_y \sigma_y. \tag{S51}$$

This Hamiltonian corresponds to a system with $d_{\max} = 1$. When $a_x = a_{xy}$, it reduces to an isotropic QBT Hamiltonian. The quantum metric for this model is given as

$$g_{xx} = \frac{2a_x^2 a_{xy}^2 k_y^2 \left(k_x^2 + k_y^2\right)^2}{\left(a_x^2\left(k_x^2 - k_y^2\right)^2 + 4a_{xy}^2 k_x^2 k_y^2\right)^2}, \quad g_{yy} = \frac{2a_x^2 a_{xy}^2 k_x^2 \left(k_x^2 + k_y^2\right)^2}{\left(a_x^2\left(k_x^2 - k_y^2\right)^2 + 4a_{xy}^2 k_x^2 k_y^2\right)^2},$$
$$g_{xy} = g_{yx} = -\frac{2a_x^2 a_{xy}^2 k_x k_y \left(k_x^2 + k_y^2\right)^2}{\left(a_x^2\left(k_x^2 - k_y^2\right)^2 + 4a_{xy}^2 k_x^2 k_y^2\right)^2}. \tag{S52}$$

A straightforward calculation leads to the optical conductivity:

$$\text{Re}[\sigma_{ij}(\omega)] = \frac{1}{16}\left(\frac{a_x}{a_{xy}} + \frac{a_{xy}}{a_x}\right)\delta_{ij}. \tag{S53}$$

Here, we consider the chemical potential $\mu = 0$ and $a_0 = 0$. When $a_x = a_{xy}$, the optical conductivity simplifies to $1/8$, consistent with the isotropic QBT result.

Under C_2 and $T = \sigma_x K$ symmetries, the most general form can be written as

$$H_{\text{aniso2}}(\boldsymbol{k}) = b_0 k^2 \sigma_0 + (b_x k_x^2 + b_y k_y^2)\sigma_x + 2b_{xy}k_x k_y \sigma_y. \tag{S54}$$

The quantum metric for this model is given as

$$g_{xx} = \frac{2b_{xy}^2 k_y^2 \left(b_x k_x^2 - b_y k_y^2\right)^2}{\left(b_x^2 k_x^4 + 2(b_x b_y + 2b_{xy}^2)^2 k_x^2 k_y^2 + b_y^2 k_y^4\right)^2}, \quad g_{yy} = \frac{2b_{xy}^2 k_x^2 \left(b_x k_x^2 - b_y k_y^2\right)^2}{\left(b_x^2 k_x^4 + 2(b_x b_y + 2b_{xy}^2)^2 k_x^2 k_y^2 + b_y^2 k_y^4\right)^2},$$
$$g_{xy} = g_{yx} = -\frac{2b_{xy}^2 k_x k_y \left(b_x k_x^2 - b_y k_y^2\right)^2}{\left(b_x^2 k_x^4 + 2(b_x a_y + 2b_{xy}^2)^2 k_x^2 k_y^2 + b_y^2 k_y^4\right)^2}. \tag{S55}$$

A straightforward calculation leads to the optical conductivity:

$$\mathrm{Re}[\sigma_{ij}(\omega)] = \delta_{ij}\frac{1}{16\pi}\int_0^{2\pi} d\theta \frac{b_{xy}^2\left(b_x - b_y + (b_x + b_y)\cos 2\theta\right)^2 \sin^2\theta}{2\left((b_x\cos^2\theta + b_y\sin^2\theta)^2 + b_{xy}^2\sin^2 2\theta\right)^2}. \tag{S56}$$

Here we consider the chemical potential $\mu = 0$ and $b_0 = 0$.

For $T = K$ case, $C_n\ \ (n = 3, 6)$ only allows the isotropic QBTs with $d_{\max} = 0$. On the other hand, $C_n\ \ (n = 2, 4)$ and $T = K$ permit the following Hamiltonian:

$$H_{\mathrm{aniso3}} = c_0 k^2 \sigma_0 + c_1(k_x^2 - k_y^2)\sigma_x + (c_2 k_x^2 + c_3 k_y^2)\sigma_z. \tag{S57}$$

The quantum metric for this model is given as

$$\begin{gathered} g_{xx} = \frac{2c_1^2(c_2+c_3)^2 k_x^2 k_y^4}{\left(c_1^2(k_x^2-k_y^2)^2 + (c_2k_x^2+c_3k_y^2)^2\right)^2},\ \ g_{yy} = \frac{2c_1^2(c_2+c_3)^2 k_x^4 k_y^2}{\left(c_1^2(k_x^2-k_y^2)^2 + (c_2k_x^2+c_3k_y^2)^2\right)^2}, \\ g_{xy} = g_{yx} = -\frac{2c_1^2(c_2+c_3)^2 k_x^3 k_y^3}{\left(c_1^2(k_x^2-k_y^2)^2 + (c_2k_x^2+c_3k_y^2)^2\right)^2}. \end{gathered} \tag{S58}$$

A straightforward calculation leads to the optical conductivity:

$$\mathrm{Re}[\sigma_{ij}(\omega)] = \delta_{ij}\frac{1}{16\pi}\int_0^{2\pi} d\theta \frac{2c_1^2(c_2+c_3)^2\cos^2\theta\sin^4\theta}{\left((c_1^2+c_2^2)\cos^4\theta - 2(c_1^2 - c_2c_3)\cos^2\theta\sin^2\theta + (c_1^2+c_3^2)\sin^4\theta\right)^2}. \tag{S59}$$

Here we consider the chemical potential $\mu = 0$ and $c_0 = 0$.

XI. INTRABAND EFFECTS

In the metallic regime near the Fermi surface, intraband transitions should be considered. The optical conductivity arising from these transitions, described in Eq. (S14), is defined as follows:

$$\sigma_{xx}^{\mathrm{intra}}(\omega) = i\int \frac{d^2k}{(2\pi)^2}\sum_n \left(-\frac{\partial f(\epsilon_{n,\boldsymbol{k}})}{\partial \epsilon_{n,\boldsymbol{k}}}\right)\frac{(v_{n,\boldsymbol{k},x})^2}{\omega + i\eta}, \tag{S60}$$

where η is a broadening factor, and $v_{n,\boldsymbol{k},x} = \partial_{k_x}\epsilon_{n,\boldsymbol{k}}$ is the group velocity along the x-direction. With finite ω, this intraband response is suppressed as $v_{n,\boldsymbol{k},x}$ becomes small, as well as when η is small. Consequently, suppression of this response is ensured by the following conditions:

- A large effective mass for ϵ_n at the Fermi level (See SFig. 2**a**).
- A small Fermi wave vector (k_F) or, equivalently, a Fermi energy close to the band-touching point (See SFig. 2**b**).
- The high cleanness of the sample (See SFig. 2**c**).

These factors collectively minimize the intraband contribution to the optical conductivity in the metallic regime.

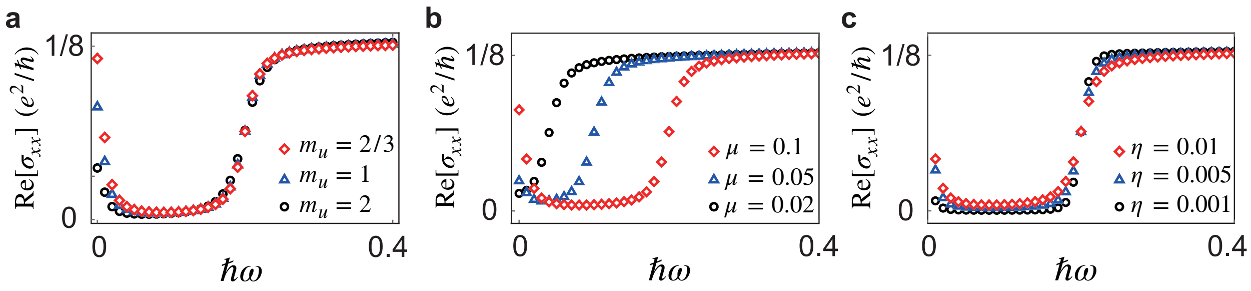

Fig. S2. **Intraband effect on optical conductivity.** **a** Optical conductivity Re[σ_{xx}] as a function of the mass of the upper band m_u. The black, blue, and red makers represent $m_u = 2, 1$, and 2/3, respectively. Here, $(\mu^*, m_l, T) = (0.2, -1, 0.003)$. **b** Chemical potential μ dependence of Re[σ_{xx}]. The black, blue, and red makers represent $\mu = 0.02, 0.05$, and 0.1, respectively. Here, $(\mu^*, m_l = -m_u, T) = (0.2, -1, 0.003)$. **c** Broadening factor dependence η dependence of Re[σ_{xx}]. The black, blue, and red makers represent $\eta = 0.001, 0.005$, and 0.01, respectively. Here, $(\mu^*, m_l = -m_u, T) = (0.2, -1, 0.003)$. In the calculations of **a-c**, we used the model in Fig. 2**b** of the main text with $d_{\max} = 1$.

XII. OPTICAL CONDUCTIVITY IN 3D

Quantum geometry influences not only the optical conductivity in 2D systems but also plays a significant role in 3D optical conductivity. In this section, we examine this effect by considering 3D isotropic quadratic band-touching models, where the energy band dispersions are identical, but the wavefunction geometries differ.

The models are defined as follows:

$$\mathcal{H}_1 = \frac{1}{2m^*}\left\{0, 0, k^2\right\} \cdot \{\sigma_x, \sigma_y, \sigma_z\} \tag{S61}$$

$$\mathcal{H}_2 = \frac{1}{2m^*}\left\{k\sqrt{k_x^2+k_y^2}, 0, kk_z\right\} \cdot \{\sigma_x, \sigma_y, \sigma_z\} \tag{S62}$$

$$\mathcal{H}_3 = \frac{1}{2m^*}\left\{kk_x, kk_y, kk_z\right\} \cdot \{\sigma_x, \sigma_y, \sigma_z\}, \tag{S63}$$

where $k^2 = k_x^2 + k_y^2 + k_z^2$. For all the models, the energy eigenvalues are the same: $\epsilon_\pm = \pm k^2/(2m^*)$. However their quantum metric tensors differ. For $\mathcal{H}_1$, $g_{ij} = 0$. For $\mathcal{H}_2$,

$$\begin{aligned} g_{xx} &= \frac{k_x^2 k_z^2}{2(k_x^2+k_y^2)k^4}, \quad g_{xy} = g_{yx} = \frac{k_x k_y k_z^2}{2(k_x^2+k_y^2)k^4}, \quad g_{yy} = \frac{k_x^2 k_z^2}{2(k_x^2+k_y^2)k^4}, \\ g_{zz} &= \frac{k_x^2+k_y^2}{2k^4}, \quad g_{xz} = g_{zx} = -\frac{k_x k_z}{2k^4}, \quad g_{yz} = g_{zy} = -\frac{k_y k_z}{2k^4}, \end{aligned} \tag{S64}$$

For $\mathcal{H}_3$,

$$\begin{aligned} g_{xx} &= \frac{k_y^2+k_z^2}{2k^4}, \quad g_{xy} = g_{yx} = -\frac{k_x k_y}{2k^4}, \quad g_{yy} = \frac{k_y^2+k_z^2}{2k^4}, \\ g_{zz} &= \frac{k_x^2+k_y^2}{2k^4}, \quad g_{xz} = g_{zx} = -\frac{k_x k_z}{2k^4}, \quad g_{yz} = g_{zy} = -\frac{k_y k_z}{2k^4}. \end{aligned} \tag{S65}$$

Similar to Eq. (S22), the three-dimensional optical conductivity for two-band systems is written as

$$\text{Re}[\sigma_{ij}(\omega)] = \frac{\pi}{2}\int \frac{d^2\boldsymbol{k}}{(2\pi)^3} F_{lu}(\boldsymbol{k})\epsilon_{ul}(\boldsymbol{k}) g_{ij}(\boldsymbol{k})\delta(\omega - \epsilon_{ul}). \tag{S66}$$

Since $\epsilon_{ul} = \epsilon_+ - \epsilon_- = k^2/m^*$, this reduces to

$$\text{RE}[\sigma_{ij}(\omega)] = \frac{1}{32\pi^2}\sqrt{m^*\omega}S_{\text{geom}}\delta_{ij}, \tag{S67}$$

where

$$S_{\text{geom}} = \int d\theta d\phi k^2 \sin\theta g_{ii}(\theta, \phi), \tag{S68}$$

where $\theta = \arccos(k_z/k)$ and $\phi = \arctan(k_y/k_x)$. Here, we consider the case where the chemical potential μ=0. Unlike the 2D case, the intensity of the optical conductivity in 3D systems depends on the mass, reflecting the significance of band dispersion. However, similar to the 2D case, the contribution from

quantum geometry is encoded in S_{geom}, which depends sorely on the wavefunction geometry. The values of S_{geom} for the above model are

$$S_{\text{geom}} = 0 \quad \text{for } \mathcal{H}_1 \tag{S69}$$

$$S_{\text{geom}} = \frac{\pi}{3} \quad \text{for } \mathcal{H}_2 \tag{S70}$$

$$S_{\text{geom}} = \frac{4\pi}{3} \quad \text{for } \mathcal{H}_3. \tag{S71}$$

Thus, despite having identical band dispersions, the models exhibit distinct optical conductivities due to differences in their wavefunction geometries.

REFERENCES

1. S. Peotta, P. Törmä, Superfluidity in topologically nontrivial flat bands. *Nat. Commun.* **6**, 8944 (2015).

2. T. Ozawa, B. Mera, Relations between topology and the quantum metric for Chern insulators. *Phys. Rev. B* **104**, 045103 (2021).

3. P. Törmä, S. Peotta, B. A. Bernevig, Superconductivity, superfluidity and quantum geometry in twisted multilayer systems. *Nat. Rev. Phys.* **4**, 528–542 (2022).

4. P. Törmä, Essay: Where can quantum geometry lead us? *Phys. Rev. Lett.* **131**, 240001 (2023).

5. Y. Onishi, L. Fu, Fundamental bound on topological gap. *Phys. Rev. X* **14**, 011052 (2024).

6. J. Yu, B. A. Bernevig, R. Queiroz, E. Rossi, P. Törmä, B.-J. Yang, Quantum geometry in quantum materials. arXiv:2501.00098 [cond-mat.mes-hall] (2024).

7. J. Yu, C. J. Ciccarino, R. Bianco, I. Errea, P. Narang, B. A. Bernevig, Non-trivial quantum geometry and the strength of electron–phonon coupling. *Nat. Phys.* **20**, 1262–1268 (2024).

8. T. Neupert, C. Chamon, C. Mudry, Measuring the quantum geometry of Bloch bands with current noise. *Phys. Rev. B* **87**, 245103 (2013).

9. N. Verma, D. Guerci, R. Queiroz, Geometric stiffness in interlayer exciton condensates. *Phys. Rev. Lett.* **132**, 236001 (2024).

10. F. Wu, S. Das Sarma, Quantum geometry and stability of moiré flatband ferromagnetism. *Phys. Rev. B* **102**, 165118 (2020).

11. I. Komissarov, T. Holder, R. Queiroz, The quantum geometric origin of capacitance in insulators. *Nat. Commun.* **15**, 4621 (2024).

12. P. M. Tam, J. Herzog-Arbeitman, J. Yu, Corner charge fluctuation as an observable for quantum geometry and entanglement in two-dimensional insulators. *Phys. Rev. Lett.* **133**, 246603 (2024).

13. Z. Han, J. Herzog-Arbeitman, B. A. Bernevig, S. A. Kivelson, Quantum geometric nesting and solvable model flat-band systems. *Phys. Rev. X* **14**, 041004 (2024).

14. Y. Fang, J. Cano, S. A. A. Ghorashi, Quantum geometry induced nonlinear transport in altermagnets. *Phys. Rev. Lett.* **133**, 106701 (2024).

15. M. Yu, P. Yang, M. Gong, Q. Cao, Q. Lu, H. Liu, S. Zhang, M. B. Plenio, F. Jelezko, T. Ozawa, N. Goldman, J. Cai, Experimental measurement of the quantum geometric tensor using coupled qubits in diamond. *Natl. Sci. Rev.* **7**, 254–260 (2020).

16. I. Amelio, N. Goldman, Lasing in non-Hermitian flat bands: Quantum geometry, coherence, and the fate of Kardar-Parisi-Zhang physics. *Phys. Rev. Lett.* **132**, 186902 (2024).

17. T. Ozawa, N. Goldman, Probing localization and quantum geometry by spectroscopy. *Phys. Rev. Res.* **1**, 032019 (2019).

18. T. Ozawa, Steady-state Hall response and quantum geometry of driven-dissipative lattices. *Phys. Rev. B* **97**, 041108 (2018).

19. J. P. Provost, G. Vallee, Riemannian structure on manifolds of quantum states. *Commun. Math. Phys.* **76**, 289–301 (1980).

20. A. Shapere, F. Wilczek, *Geometric Phases in Physics* (World Scientific, 1989), vol. **5**.

21. Y.-Q. Ma, S. Chen, H. Fan, W.-M. Liu, Abelian and non-Abelian quantum geometric tensor. *Phys. Rev. B* **81**, 245129 (2010).

22. S. Matsuura, S. Ryu, Momentum space metric, nonlocal operator, and topological insulators. *Phys. Rev. B* **82**, 245113 (2010).

23. N. Nagaosa, J. Sinova, S. Onoda, A. H. MacDonald, N. P. Ong, Anomalous Hall effect. *Rev. Mod. Phys.* **82**, 1539–1592 (2010).

24. D. Xiao, M.-C. Chang, Q. Niu, Berry phase effects on electronic properties. *Rev. Mod. Phys.* **82**, 1959–2007 (2010).

25. L. Liang, T. I. Vanhala, S. Peotta, T. Siro, A. Harju, P. Törmä, Band geometry, Berry curvature, and superfluid weight. *Phys. Rev. B* **95**, 024515 (2017).

26. J.-W. Rhim, K. Kim, B.-J. Yang, Quantum distance and anomalous Landau levels of flat bands. *Nature* **584**, 59–63 (2020).

27. Y. Hwang, J.-W. Rhim, B.-J. Yang, Geometric characterization of anomalous Landau levels of isolated flat bands. *Nat. Commun.* **12**, 6433 (2021).

28. W. J. Jankowski, J. J. P. Thompson, B. Monserrat, R.-J. Slager, Excitonic topology and quantum geometry in organic semiconductors. arXiv:2406.11951 [cond-mat.mes-hall] (2024).

29. X. Hu, T. Hyart, D. I. Pikulin, E. Rossi, Quantum-metric-enabled exciton condensate in double twisted bilayer graphene. *Phys. Rev. B* **105**, L140506 (2022).

30. R. Resta, Polarization fluctuations in insulators and metals: New and old theories merge. *Phys. Rev. Lett.* **96**, 137601 (2006).

31. N. Verma, R. Queiroz, Quantum metric in step response. arXiv:2406.17845 [cond-mat.mes-hall] (2024).

32. A. Bouhon, A. Timmel, R.-J. Slager, Quantum geometry beyond projective single bands. arXiv:2303.02180 [cond-mat.mes-hall] (2023).

33. C.-g. Oh, J.-W. Rhim, B.-J. Yang, Revisiting the magnetic responses of bilayer graphene from the perspective of quantum distance. *Phys. Rev. B* **110**, 155412 (2024).

34. C.-g. Oh, K. W. Kim, J.-W. Rhim, Thermoelectric transport driven by the Hilbert–Schmidt distance. *Adv. Sci.* **11**, 2411313 (2024).

35. C.-g. Oh, D. Cho, S. Y. Park, J.-W. Rhim, Bulk-interface correspondence from quantum distance in flat band systems. *Commun. Phys.* **5**, 320 (2022).

36. H. Kim, C.-g. Oh, J.-W. Rhim, General construction scheme for geometrically nontrivial flat band models. *Commun. Phys.* **6**, 305 (2023).

37. A. M. Cook, B. M. Fregoso, F. De Juan, S. Coh, J. E. Moore, Design principles for shift current photovoltaics. *Nat. Commun.* **8**, 14176 (2017).

38. F. De Juan, A. G. Grushin, T. Morimoto, J. E. Moore, Quantized circular photogalvanic effect in Weyl semimetals. *Nat. Commun.* **8**, 15995 (2017).

39. T. Holder, D. Kaplan, B. Yan, Consequences of time-reversal-symmetry breaking in the light-matter interaction: Berry curvature, quantum metric, and diabatic motion. *Phys. Rev. Res.* **2**, 033100 (2020).

40. P. Bhalla, K. Das, D. Culcer, A. Agarwal, Resonant second-harmonic generation as a probe of quantum geometry. *Phys. Rev. Lett.* **129**, 227401 (2022).

41. B. Ghosh, Y. Onishi, S.-Y. Xu, H. Lin, L. Fu, A. Bansil, Probing quantum geometry through optical conductivity and magnetic circular dichroism. *Sci. Adv.* **10**, eado1761 (2024).

42. M. Ezawa, Analytic approach to quantum metric and optical conductivity in Dirac models with parabolic mass in arbitrary dimensions. *Phys. Rev. B* **110**, 195437 (2024).

43. J. Ahn, G.-Y. Guo, N. Nagaosa, A. Vishwanath, Riemannian geometry of resonant optical responses. *Nat. Phys.* **18**, 290–295 (2022).

44. D. Xiao, W. Yao, Q. Niu, Valley-contrasting physics in graphene: Magnetic moment and topological transport. *Phys. Rev. Lett.* **99**, 236809 (2007).

45. J. R. Schaibley, H. Yu, G. Clark, P. Rivera, J. S. Ross, K. L. Seyler, W. Yao, X. Xu, Valleytronics in 2D materials. *Nat. Rev. Mater.* **1**, 16055 (2016).

46. T. Ohta, A. Bostwick, T. Seyller, K. Horn, E. Rotenberg, Controlling the electronic structure of bilayer graphene. *Science* **313**, 951–954 (2006).

47. H. Min, B. Sahu, S. K. Banerjee, A. H. MacDonald, Ab initiotheory of gate induced gaps in graphene bilayers. *Phys. Rev. B* **75**, 155115 (2007).

48. E. McCann, M. Koshino, The electronic properties of bilayer graphene. *Rep. Prog. Phys.* **76**, 056503 (2013).

49. M. Kang, L. Ye, S. Fang, J.-S. You, A. Levitan, M. Han, J. I. Facio, C. Jozwiak, A. Bostwick, E. Rotenberg, B. J. Yang, J. G. Checkelsky, R. Comin, Dirac fermions and flat bands in the ideal kagome metal FeSn. *Nat. Mater.* **19**, 163–169 (2020).

50. M. Han, H. Inoue, S. Fang, C. John, L. Ye, M. K. Chan, D. Graf, T. Suzuki, M. P. Ghimire, W. J. Cho, J. G. Checkelsky, R. Comin, Evidence of two-dimensional flat band at the surface of antiferromagnetic kagome metal FeSn. *Nat. Commun.* **12**, 5345 (2021).

51. Z. Sun, H. Zhou, C. Wang, S. Kumar, D. Geng, S. Yue, X. Han, Y. Haraguchi, K. Shimada, P. Cheng, J. Lan, S. Huang, J. Guan, T. R. Chang, Z. Shi, Y. Shi, Y. Yao, L. Ye, J. G. Checkelsky, R. Comin, K. Ibrahim, Observation of topological flat bands in the kagome semiconductor Nb3Cl8. *Nano Lett.* **22**, 4596–4602 (2022).

52. J. H. Lee, G. W. Kim, I. Song, Y. Kim, Y. Lee, S. J. Yoo, D.-Y. Cho, J.-W. Rhim, J. Jung, G. Kim, H. W. Yeom, Atomically thin two-dimensional kagome flat band on the silicon surface. *ACS Nano* **18**, 25535–25541 (2024).

53. J. Jung, H. Lim, B.-J. Yang, Quantum geometry and landau levels of quadratic band crossings. *Phys. Rev. B* **109**, 035134 (2024).

54. J.-W. Rhim, B.-J. Yang, Classification of flat bands according to the band-crossing singularity of Bloch wave functions. *Phys. Rev. B* **99**, 045107 (2019).

55. Y. Hwang, J. Jung, J.-W. Rhim, B.-J. Yang, Wave-function geometry of band crossing points in two dimensions. *Phys. Rev. B* **103**, L241102 (2021).

56. W. J. Jankowski, A. S. Morris, A. Bouhon, F. N. Ünal, R.-J. Slager, Optical manifestations of topological Euler class. arXiv:2311.07545 [cond-mat.mes-hall] (2023).

57. W. J. Jankowski, R.-J. Slager, Quantized integrated shift effect in multigap topological phases. *Phys. Rev. Lett.* **133**, 186601 (2024).

58. L. Zhu, S.-S. Wang, S. Guan, Y. Liu, T. Zhang, G. Chen, S. A. Yang, Blue phosphorene oxide: Strain-tunable quantum phase transitions and novel 2D emergent fermions. *Nano Lett.* **16**, 6548–6554 (2016).

59. S.-S. Wang, Y. Liu, Z.-M. Yu, X.-L. Sheng, L. Zhu, S. Guan, S. A. Yang, Monolayer Mg2C: Negative Poisson's ratio and unconventional two-dimensional emergent fermions. *Phys. Rev. Mater.* **2**, 104003 (2018).

60. S. Park, S. Kang, H. Kim, K. H. Lee, P. Kim, S. Sim, N. Lee, B. Karuppannan, J. Kim, J. Kim, K. I. Sim, M. J. Coak, Y. Noda, C.-H. Park, J. H. Kim, J.-G. Park, Kagome van-der-waals $Pd_3P_2S_8$ with flat band. *Sci. Rep.* **10**, 20998 (2020).

61. H. Min, A. H. MacDonald, Origin of universal optical conductivity and optical stacking sequence identification in multilayer graphene. *Phys. Rev. Lett.* **103**, 067402 (2009).

62. Y. Wang, Z. Ni, L. Liu, Y. Liu, C. Cong, T. Yu, X. Wang, D. Shen, Z. Shen, Stacking-dependent optical conductivity of bilayer graphene. *ACS Nano* **4**, 4074–4080 (2010).

63. F. Yang, L. Miao, Z. F. Wang, M.-Y. Yao, F. Zhu, Y. R. Song, M.-X. Wang, J.-P. Xu, A. V. Fedorov, Z. Sun, G. B. Zhang, C. Liu, F. Liu, D. Qian, C. L. Gao, J.-F. Jia, Spatial and energy distribution of topological edge states in single Bi(111) bilayer. *Phys. Rev. Lett.* **109**, 016801 (2012).

64. K. S. Novoselov, A. K. Geim, S. V. Morozov, D. Jiang, M. I. Katsnelson, I. V. Grigorieva, S. V. Dubonos, A. A. Firsov, Two-dimensional gas of massless Dirac fermions in graphene. *Nature* **438**, 197–200 (2005).

65. L. A. Falkovsky, A. A. Varlamov, Space-time dispersion of graphene conductivity. *Eur. Phys. J. B.* **56**, 281–284 (2007).

66. R. R. Nair, P. Blake, A. N. Grigorenko, K. S. Novoselov, T. J. Booth, T. Stauber, N. M. R. Peres, A. K. Geim, Fine structure constant defines visual transparency of graphene. *Science* **320**, 1308 (2008).

67. G. Kresse, J. Furthmüller, Efficiency of ab-initio total energy calculations for metals and semiconductors using a plane-wave basis set. *Comput. Mater. Sci.* **6**, 15–50 (1996).

68. G. Kresse, J. Furthmüller, Efficient iterative schemes for ab initio total-energy calculations using a plane-wave basis set. *Phys. Rev. B* **54**, 11169–11186 (1996).

69. P. E. Blöchl, Projector augmented-wave method. *Phys. Rev. B* **50**, 17953–17979 (1994).

70. J. P. Perdew, K. Burke, M. Ernzerhof, Generalized gradient approximation made simple. *Phys. Rev. Lett.* **77**, 3865–3868 (1996).

71. Q. Wang, X.-L. Qiu, C. Pei, B.-C. Gong, L. Gao, Y. Zhao, W. Cao, C. Li, S. Zhu, M. Zhang, Y. Chen, K. Liu, Y. Qi, Superconductivity emerging from a pressurized van der waals kagome material $Pd_3P_2S_8$. *New J. Phys.* **25**, 043001 (2023).

72. T. Nagao, J. T. Sadowski, M. Saito, S. Yaginuma, Y. Fujikawa, T. Kogure, T. Ohno, Y. Hasegawa, S. Hasegawa, T. Sakurai, Nanofilm allotrope and phase transformation of ultrathin Bi film on Si(111) –7 × 7. *Phys. Rev. Lett.* **93**, 105501 (2004).

73. G. Pizzi, V. Vitale, R. Arita, S. Blügel, F. Freimuth, G. Géranton, M. Gibertini, D. Gresch, C. Johnson, T. Koretsune, J. Ibañez-Azpiroz, H. Lee, J.-M. Lihm, D. Marchand, A. Marrazzo, Y. Mokrousov, J. I. Mustafa, Y. Nohara, Y. Nomura, L. Paulatto, S. Poncé, T. Ponweiser, J. Qiao, F. Thöle, S. S. Tsirkin, M. Wierzbowska, N. Marzari, D. Vanderbilt, I. Souza, A. A. Mostofi, J. R. Yates, Wannier90 as a community code: new features and applications. *J. Phys. Condens. Matter* **32**, 165902 (2020).

74. C. Fang, M. J. Gilbert, X. Dai, B. A. Bernevig, Multi-weyl topological semimetals stabilized by point group symmetry. *Phys. Rev. Lett.* **108**, 266802 (2012).
